# Supplementary material for: Evaluating the Impacts of Climate Factors and Flavonoids Content on Chinese Prickly Ash Peel Color Based on HPLC-MS and Structural Equation Model
Source: Foods. 2022 Aug 22;11(16):2539. doi: 10.3390/foods11162539 (PMC9407495; doi:10.3390/foods11162539)
Supplement: Supplementary file 1 [file foods-11-02539-s001.zip › foods-1854872-Supplementary.pdf]

Supplement materials

Title

Evaluating the impacts of climate factors and flavonoids compounds on Chinese prickly ash peels color based on HPLC-MS and structural equation model

Author

Tao Zheng[1], Ding-ling Zhang[1*], Bing-yin Sun[2], Shu-ming Liu[1*]

[1] Northwest Agriculture and Forestry University, College of Science, Yangling 712100, China;

lxyzt@nwafu.edu.cn (T.Z.); zhangdl04@126.com (D. Z.), zhengtyhy@163.com (S. L.)

[2] Yangling Vocational &Technical College, Yangling 712100, China; sunby001229@sina.com

*Corresponding author

+E-mail: zhangdl04@126.com, zhengtyhy@163.com

Table S1. Chinese prickly ash peels geographic information of sampling localities.

Provenance	Species	Latitude	Longtitude	Elevation(m)	Location	Regions
A1	*Zanthoxylum bungeanum* Maxim	36°03'11"	101°23'20"	2188	Guide Qinghai	Northwest China
A2	*Zanthoxylum bungeanum* Maxim	35°52'59"	102°26'56"	1897	Xunhua Qinghai	Northwest China
A3	*Zanthoxylum bungeanum* Maxim	35°49'58"	102°31'39"	1857	Xunhua Qinghai	Northwest China
A4	*Zanthoxylum bungeanum* Maxim	29°41'04"	102°28'13"	1791	Hanyuan Sichuan	Southwest China
A5	*Zanthoxylum bungeanum* Maxim	29°41'34’’	102°28'43"	1869	Hanyuan Sichuan	Southwest China
A6	*Zanthoxylum bungeanum* Maxim	33°30'15"	103°57'36"	2007	Jiuzhaigou Sichuan	Southwest China
B1	*Zanthoxylum bungeanum* Maxim	33°02'54"	104°39'54"	1856	Wenxian Gansu	Northwest China
B2	*Zanthoxylum bungeanum* Maxim	33°30'18"	105°04’30"	1420	Wudu Gansu	Northwest China
B3	*Zanthoxylum bungeanum* Maxim	34°53'22"	105°33'58"	1621	Qinan Gansu	Northwest China
B4	*Zanthoxylum bungeanum* Maxim	34°53'18"	105°33'53"	1646	Qinan Gansu	Northwest China
B5	*Zanthoxylum bungeanum* Maxim	33°59'06"	106°39'24"	1011	Fengxian Shaanxi	Northwest China
C1	*Zanthoxylum bungeanum* Maxim	34°59'15"	109°13'41"	787	Fuping Shaanxi	Northwest China
D1	*Zanthoxylum bungeanum* Maxim	37°45’17"	109°43'58"	1195	Hengshan Shaanxi	Northwest China
C2	*Zanthoxylum bungeanum* Maxim	35°24'45"	110°14'36"	866	Hancheng Shaanxi	Northwest China
C3	*Zanthoxylum bungeanum* Maxim	35°24'31"	110°14'44"	880	Hancheng Shaanxi	Northwest China
C4	*Zanthoxylum bungeanum* Maxim	34°56'21"	110°23'39"	378	Yongji Shanxi	North China
C5	*Zanthoxylum bungeanum* Maxim	34°56'21"	110°23'39"	378	Yongji Shanxi	North China
C6	*Zanthoxylum bungeanum* Maxim	34°31'07"	110°34'16"	540	Lingbao Henan	Central China
C7	*Zanthoxylum bungeanum* Maxim	34°31'47"	110°34'32"	545	Lingbao Henan	Central China
D2	*Zanthoxylum bungeanum* Maxim	37°33'49"	112°07'11"	847	Jiaocheng Shanxi	North China
D3	*Zanthoxylum bungeanum* Maxim	36°34'41"	113°50'32"	617	Shexian Hebei	North China
E1	*Zanthoxylum bungeanum* Maxim	35°02'59"	117°35'09"	201	Zaozhuang Shandong	East China
E2	*Zanthoxylum bungeanum* Maxim	35°02'59"	117°35'09"	201	Zaozhuang Shandong	East China
E3	*Zanthoxylum bungeanum* Maxim	35°02'59"	117°35'09"	201	Zaozhuang Shandong	East China
E4	*Zanthoxylum bungeanum* Maxim	36°30'08"	117°35'19"	330	Laiwu Shandong	East China
E5	*Zanthoxylum bungeanum* Maxim	36°30'08"	117°35'19"	330	Laiwu Shandong	East China

Table S2. Data on the climate factors.

Location	X_{AMT} (°C)	X_{AMAT} (°C)	X_{AMIT} (°C)	X_{RH} (%)	X_{AAP} (mm)	X_{MW} (m/s)	X_{MAW}(m/s)	X_{EW} (m/s)	X_{ASD}(h)	X_{ASP} (%)
Guide	8.94	16.14	3.10	46.55	266.60	1.84	8.38	15.77	2531.40	58.14
Xunhua	10.08	16.67	4.81	47.80	270.30	3.06	12.47	18.66	2470.90	56.64
Hanyuan	16.24	20.62	13.48	69.48	930.80	2.24	11.23	17.27	1123.50	25.91
Jiuzhaigou	13.10	19.00	8.50	64.30	813.90	1.87	6.80	10.90	1613.40	45.00
Wenxian	15.10	20.20	11.10	62.00	799.30	1.86	10.20	16.10	1725.80	39.46
Wudu	15.45	20.95	11.53	56.35	785.90	1.63	8.81	15.90	1623.60	37.02
Qinan	11.64	18.08	7.14	70.44	591.40	1.23	6.22	12.04	1714.40	39.08
Fengxian	12.26	18.98	7.68	71.79	720.40	1.64	8.05	12.58	2109.35	47.70
Fuping	14.12	20.05	9.31	67.06	597.30	1.86	7.04	11.40	1247.40	26.18
Hengshan	10.10	17.42	3.81	51.98	307.00	2.16	10.81	18.48	3235.70	73.85
Hancheng	14.14	20.21	9.30	61.38	509.80	1.47	7.51	14.47	2384.70	54.09
Yongjing	14.99	21.00	10.23	65.55	540.50	2.38	9.04	14.78	2349.50	52.56
Lingbao	14.83	20.27	10.49	61.80	561.50	2.46	9.26	14.38	1932.60	43.80

Jiaocheng	11.42	19.01	4.81	59.85	500.00	1.69	9.48	17.09	2344.30	53.20
Shexian	14.38	20.96	9.53	55.84	653.40	1.26	6.47	13.22	2129.00	48.32
Zaozhaung	15.72	21.03	11.29	66.31	1017.70	1.77	7.06	13.23	1711.50	38.92
Laiwu	14.58	19.79	10.18	62.75	803.20	1.67	7.78	15.63	2151.30	48.84

Note: Data are averages of replications. X_{AMT} (°C)-Annual mean temperature, X_{AMAT} (°C)-Annual mean maximum temperature, X_{AMIT} (°C)-Annual mean minimum temperature, X_{RH} (%)-Annual relative humidity, X_{AAP} (mm)-Annual average precipitation, X_{MW} (m/s)-Mean wind speed, X_{MAW} (m/s)-Maximum wind speed, X_{EW} (m/s)-Extreme wind speed, X_{ASD} (h)-Annual sunshine duration and X_{ASP} (%)-Percentage of sunshine.

Table S3. UPLC-MS/MS information of 15 flavonoids.

Compounds	Q1 (Da)	Molecular Weight (Da)	Ionization model	Formula
Hyperoside	4.63E+02	4.64E+02	[M-H]-	C21H20O12
Quercitrin	4.49E+02	4.48E+02	[M+H]+	C21H20O11
Catechin	2.89E+02	2.90E+02	[M-H]-	C15H14O6
Hesperetin	3.01E+02	3.02E+02	[M-H]-	C16H14O6
Rutin	6.09E+02	6.10E+02	[M-H]-	C27H30O16
Kaempferol	2.85E+02	2.86E+02	[M-H]-	C15H10O6
Peonidin O-hexoside	4.63E+02	4.63E+02	[M]+	C22H23O11+
Cyanidin 3-O-glucoside	4.49E+02	4.49E+02	[M]+	C21H21O11+
Cyanidin O-syringic acid	4.65E+02	4.67E+02	[M-2H]-	C24H19O10+
Luteolin	2.85E+02	2.86E+02	[M-H]-	C15H10O6
Quercetin	3.03E+02	3.02E+02	[M+H]+	C15H10O7
Cyanidin 3-O-galactoside	4.49E+02	4.49E+02	[M]+	C21H21O11+
Peonidin 3-O-glucoside	4.63E+02	4.63E+02	[M]+	C22H23O11+
Chlorogenic acid	3.53E+02	3.54E+02	[M-H]-	C16H18O9
Apigenin	4.33E+02	4.32E+02	[M+H]+	C15H10O5

Table S4. Correlation analysis between climate factors.

Climate	X_{AMT}	X_{AMAT}	X_{AMIT}	X_{RH}	X_{AAP}	X_{MW}	X_{MAW}	X_{EW}	X_{ASD}	X_{ASP}
X_{AMT}	1.000	0.958**	0.980**	0.592**	0.793**	-0.139	-0.204	-0.247	-0.613**	-0.637**
X_{AMAT}	0.958**	1.000	0.895**	0.574**	0.714**	-0.238	-0.308	-0.321	-0.484*	-0.517**
X_{AMIT}	0.980**	0.895**	1.000	0.623**	0.810**	-0.112	-0.153	-0.230	-0.702**	-0.719**
X_{RH}	0.592**	0.574**	0.623**	1.000	0.677**	-0.409*	-0.463*	-0.601**	-0.650**	-0.661**
X_{AAP}	0.793**	0.714**	0.810**	0.677**	1.000	-0.353	-0.357	-0.371	-0.728**	-0.710**
X_{MW}	-0.139	-0.238	-0.112	-0.409*	-0.353	1.000	0.827**	0.593**	0.186	0.188
X_{MAW}	-0.204	-0.308	-0.153	-0.463*	-0.357	0.827**	1.000	0.885**	0.213	0.202
X_{EW}	-0.247	-0.321	-0.230	-0.601**	-0.371	0.593**	0.885**	1.000	0.408*	0.380
X_{ASD}	-0.613**	-0.484*	-0.702**	-0.650**	-0.728**	0.186	0.213	0.408*	1.000	0.988**
X_{ASP}	-0.637**	-0.517**	-0.719**	-0.661**	-0.710**	0.188	0.202	0.380	0.988**	1.000

Note: Data are averages of replications. X_{AMT} (°C)-Annual mean temperature, X_{AMAT} (°C)-Annual mean maximum temperature, X_{AMIT} (°C)-Annual mean minimum temperature, X_{RH} (%)-Annual relative humidity, X_{AAP} (mm)-Annual average precipitation, X_{MW} (m/s)-Mean wind speed, X_{MAW} (m/s)-Maximum wind speed, X_{EW} (m/s)-Extreme wind speed, X_{ASD} (h)-Annual sunshine duration and X_{ASP} (%)-Percentage of sunshine. ** represents significant correlation at P=0.01 level, * significant correlation at P=0.05 level.

Table S5. The regressive equation between climate factors and effective compounds of Chinese prickly ash peels.

Compounds	Regression	R	R^2	F	P

Y_{HY}	$y= -0.452X_{RH}^{**}+ 42.010$	0.720	0.518	25.811	**
Y_{LU}	$y= -0.006X_{ASD} + 0.245X_{EW} + 0.196X_{ASP}+0.378$	0.798	0.637	6.450	**
Y_{QI}	$y= -3.188X_{AMAT}^{*} - 0.076X_{ASD} +2.894X_{ASP}+2.080X_{MAW}^{*}+ 83.115$	0.902	0.814	23.032	*
Y_{CA}	$y=1.348X_{MAW}^{*} - 0.035X_{ASD} +1.381 X_{ASP} + 3.536$	0.748	0.559	9.310	*
Y_{QU}	$y= -1.628X_{AMT}+ 1.307X_{AMIT} +12.348$	0.766	0.587	4.952	*
Y_{AP}	$y= -0.458X_{AMAT}^{*} - 0.001X_{ASD} + 13.651$	0.794	0.630	12.362	**
Y_{PH}	$y= -0.641X_{AMAT}^{*}- 0.002X_{ASD} +0.567X_{MAW} -1.408X_{MW}+19.972$	0.839	0.704	12.465	**
Y_{PG}	$y= -0.739X_{AMAT}^{**} - 0.002X_{ASD} +0.260X_{MAW}^{*} +21.100$	0.799	0.639	12.982	**
Y_{CGC}	$y= -0.624X_{AMAT}^{*}+ 20.828$	0.776	0.602	7.004	*
Y_{CSA}	$y= -0.725 X_{AMAT}^{**} +22.594$	0.759	0.576	10.899	**
Y_{CGT}	$y= 2.551X_{AMIT} -3.550 X_{AMT} +32.091$	0.805	0.649	21.219	**
Y_{L*}	$y=-2.574 X_{AMAT}^{**}+133.355$	0.829	0.687	14.947	**
Y_{a*}	$y=-6.267X_{AMAT}^{**}-0.710X_{ASP}+2.214X_{MAW}^{*}+257.315$	0.812	0.660	6.323	**
Y_{b*}	$y=4.968X_{AMAT}^{*}-2.138X_{AMT}^{*}+0.015X_{AAP}+33.416$	0.840	0.705	10.347	**

Note: ** represents significant correlation at $P<0.01$ level, * significant correlation at $P<0.05$ level; Y_{HY}-Hyperoside, Y_{LU}-Luteolin, Y_{KP}-Kaempferol, Y_{QI}-Quercitrin, Y_{C}-Catechin, Y_{RU}-Rutin, Y_{CA}-Chlorogenic acid, Y_{QU}-Quercetin, Y_{HE}-Hesperetin, Y_{AP}-Apigenin, Y_{PH}-Peonidin O-hexoside, Y_{PG}-Peonidin 3-O-glucoside, Y_{CGC}-Cyanidin 3-O-glucoside, Y_{CSA}-Cyanidin O-syringic acid, Y_{CGT}-Cyanidin 3-O-galactoside. X_{AMT} (°C)-Annual mean temperature, X_{AMAT} (°C)-Annual mean maximum temperature, X_{AMIT} (°C)-Annual mean minimum temperature, X_{RH} (%)-Annual relative humidity, X_{AAP} (mm)-Annual average precipitation, X_{MW} (m/s)-Mean wind speed, X_{MAW} (m/s)-Maximum wind speed, X_{EW} (m/s)-Extreme wind speed, X_{ASD} (h)-Annual sunshine duration and X_{ASP} (%)-Percentage of sunshine.
